# Effect of mechanical treatment from extrusion process on physicochemical properties of okara cellulose powder

**DOI:** 10.1038/s41598-024-73819-5

**Published:** 2024-09-27

**Authors:** Hataichanok Kantrong, Aunchalee Aussanasuwannakul, Natita Rodkwan, Wanida Tewaruth Chitisankul

**Affiliations:** 1 Department of Food Processing and Preservation, Institute of Food Research and Product Development, Kasetsart University, Chatuchak, Bangkok, 10900 Thailand; 2 Department of Food Chemistry and Physics, Institute of Food Research and Product Development, Kasetsart University, Chatuchak, Bangkok, 10900 Thailand; 3 Department of Nutrition and Health, Institute of Food Research and Product Development, Kasetsart University, Chatuchak, Bangkok, 10900 Thailand

**Keywords:** Extrusion, Mechanical treatment, Okara powder, Engineering, Materials science

## Abstract

Okara is a by-product obtained from soybean milk containing high dietary fiber. This study focused on how to add value to okara by converting it into okara cellulose powder. The feasibility of using mechanical energy from the extrusion process to produce cellulose powder from okara was compared with the traditional mechanical method, hydrothermal and untreated okara powder (SBM). The extrusion process was carried out at different screw speeds (350–450 rpm), amount of water (0.5–1 L per hour), and number of treatment cycles. The physicochemical and functional properties of the okara cellulose powder and specific mechanical energy were analyzed. Results showed that the particle size of samples treated by 6 cycles of extrusion reduced to three times lower than SBM. The sample subjected to hydrothermal and extrusion showed significantly increased swelling ratio, water solubility index (WSI), and antioxidant capacity. However, only the extrusion treatment could improve the rheological properties. Increasing the cycles of extrusion treatment increased the swelling ratio and WSI values. Extrusion altered the color and chemical composition by decreasing the lightness and total dietary fiber. Extrusion treatment was proved to be an effective mechanical method for improving okara properties and converting the by-product into a potentially value-added ingredient for use in future food applications.

## Introduction

Okara or soybean paste is a by-product obtained from soybean milk or tofu production. Each 1 kg of soy milk or tofu product generates 1.1–1.2 kg of soybean paste, which is mostly used as animal feed or burnt as waste in Asian countries. However, okara contains nutritional benefits as 15.2–33.4% protein content (dry matter), is rich in dietary fiber (about 55%), and contains phytochemical components such as cellulose and hemicellulose^1–3^.

Dietary fiber obtained from agricultural products can be used to extract cellulose for various usages. Traditionally, dietary fiber has been used in the textile, paper, and fine chemical industries; however, recently, other potential applications include the food, agricultural, pharmaceutical, medical, environmental, and energy industries^3^. Okara is also a favored ingredient selected to modify food properties because it is a good source of fiber. Three methods are commonly used to extract cellulose as chemical, enzymatic, and mechanical treatments. Chemical treatments typically involve acid hydrolysis and provide good cellulose structure, physicochemical, and functional properties. Some disadvantages have been reported including chemical degradation of the cellulose, corrosion of the processing equipment, environmental damage, time-consuming process, high cost, and safety issues. Enzymatic treatment methods use enzymes to hydrolyze the cellulose and are safer and more environmentally friendly than chemical methods. Enzymatic treatments are time-consuming, with high contamination and cost^3,4^. Mechanical treatment methods involve creating shear forces under high pressure using a high-pressure homogenizer, and sonication or high-frequency sound waves to break up the molecules of substances or cells^3^. Extrusion processes^5–7^, wet-grinding^1,8^, and ball milling have also been used. Industrial-scale cellulose production generally utilizes chemical and mechanical methods to reduce energy requirements. Mechanical methods used to produce nanocellulose on an industrial scale include commercial stone grinding, ultra-fine friction grinding, and microfluidization^9^, while using extrusion processes to extract cellulose on an industrial scale is not popular. Previous research indicated the high potential of the extrusion process to produce cellulose^5–7,10^.

Many reports have detailed the preparation of cellulose using the extrusion process. In 2015, Hu and colleagues^10^studied the nanofibrillation of pulp fibers by twin-screw extrusion. Refined bleached kraft pulp was passed through a twin-screw extruder (TSE) several times at a high concentration of 28 wt% solid content. Results showed that the fibrillation degree of the pulp was enhanced with a higher number of passes. The optimal compromise between fibrillation degree and acceptable degradation occurred at 3, 4, 5, 8, and 14 passes, with the number of passes selected depending on the quality requirement for cellulose fiber application. Banvillet et al.^7^ studied the deconstruction of cellulose fibers using twin-screw extrusion and enzymatic hydrolysis via bioextrusion for cellulose nanofibril (CNF) production. Results showed that the bioextrusion process increased the crystallinity index and produced CNF with little or no fiber aggregation, resulting in better properties. Enzymes were recovered in this process by centrifugation, and this technology consumed less energy than traditional methods.

The papers reviewed above suggested that using an extrusion process could result in an effective green technology to produce okara cellulose powder. Therefore, this study evaluated the feasibility of using extrusion to produce cellulose powder from okara on a commercial scale to add value to this waste by-product. Extrusion is a low-cost process with high production capacity that reduces the use of chemicals and is environmentally friendly. The extrusion process parameters studied were screw speed, amount of water added to the system, and number of treatments. The physical, chemical, and functional properties of the okara cellulose powder were also determined.

## Materials and methods

### Preparation of okara powder

Okara, as a by-product from soybean milk processing, was collected from a pilot plant in the Institute, dried using a hot air oven at 80 °C for 8 h, and then ground using a Pin Mill (Alpine Augsburg, Germany). The final moisture content was 6.29 ± 0.02% w.b.

### Traditional treatment or hydrothermal extraction (adopted from Yingkamhaeng and Sukya^11^)

Two hundred and fifty grams of okara paste obtained from soy milk processing was placed in a glass bottle and 50 mL, of water was added before covering with a lid. The hydrothermal process was applied using a water spray retort (RCS-60SPXTG, Hisaka Works, Japan) at 121 °C for 30 min, and then dried using a drum dryer at 140 °C with drum speed 7 rpm. Finally, the treated sample was ground using a Pin Mill.

### Extrusion process

**Fig. 1:**
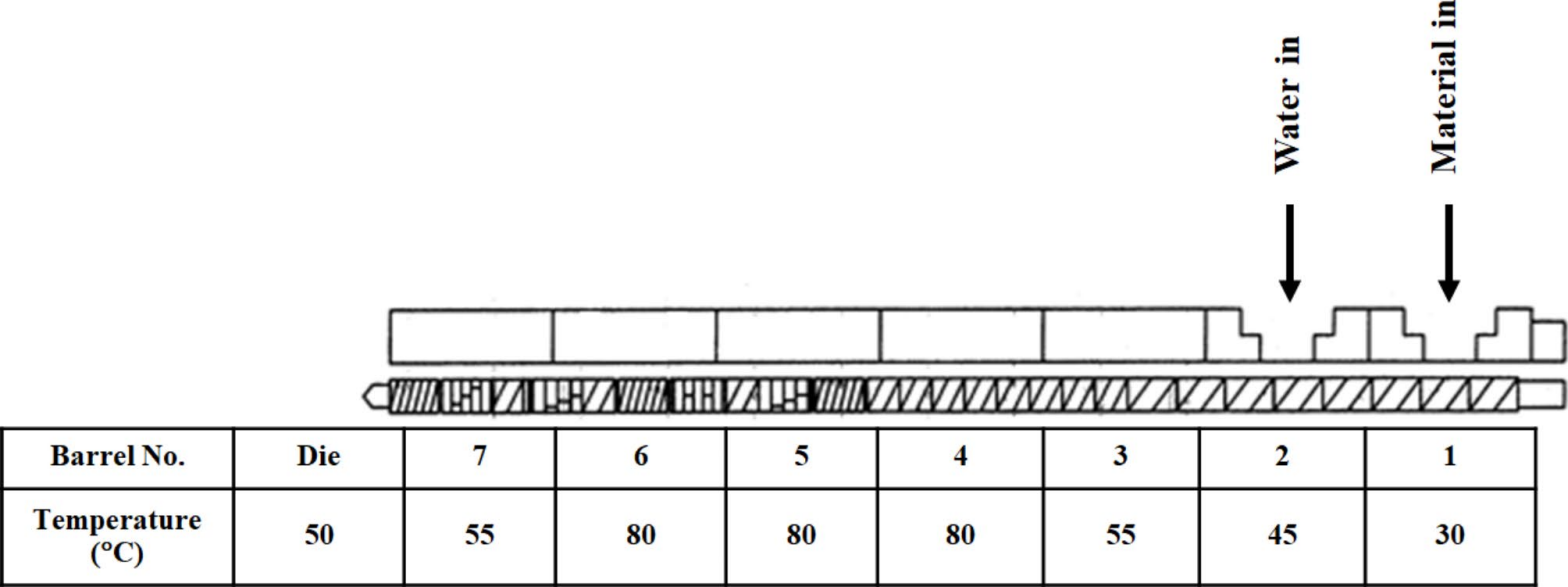
Screw and temperature extrusion profiles.

The okara powder obtained from “Preparation of okara powder” was processed using an intermeshing co-rotating twin-screw extruder (Hermann Berstorff Laboratory, ZE25 × 33D model, Germany) with L/D ratio 870:25. Process parameters studied in this research were amount of water added in the system (FM) varied at 0.5 and 1 L per hour, screw speed (SS) (350 and 450 rpm), and number of treatments (T) at 1 and 3 times. The maximum temperature of the barrel (H4-6) was maintained at 80 °C because increasing the temperature above this value caused burns. The temperature profile is exhibited in Fig. 1. The extrudate was dried in a hot air oven at 80 °C for 30 min and then reground using a Pin Mill.

### Determination of physical properties

#### Particle size

Mean particle size was measured using a laser diffraction particle size analyzer (Malvern Panalytical Ltd, Mastersizer 3000, UK). Five hundred milliliters of distilled water were used as a dispersant to suspend the sample (5 g) in the circular chamber (Hydro EV, UK), and the particle size was calculated and determined as mean value (D[3,2] from ).

#### Color

The color of the okara powder was analyzed using the CIE color system (L*, a* and b*) by a colorimeter (HunterLab, ColorQuest XE, Reston, VA), where L* is lightness, a* is redness, and b* is yellowness. The calorimeter was calibrated before use by a standard white tile for each treatment.

#### Swelling properties

The method used was adopted from Wu et al.^3^. One gram of sample (M1) was weighed into a 50 mL centrifuge tube, and then added with 35 mL of water and shaken well. The mixture was left to stand at room temperature for 24 h and then centrifuged at 3000 rpm for 15 min. The clear fraction was then separated from the precipitate which was weighed (M2). The swelling ratio was calculated using the equation below:

$$\:Swelling\:ratio=\:\frac{({M}_{2}-{M}_{1})}{{M}_{1}}$$

#### Water solubility index, WSI^12,13^

The sample (2.5 g) was dissolved in warm water in a 50 mL centrifuge tube and stirred well for 30 min before centrifuging at 3000*g* for 30 min to separate the clear and gel fractions. The separated clear fraction was evaporated in a water bath and then dried in a vacuum oven at 70 °C until the weight was constant. Finally, the WSI was determined using the equation.

$$\:WSI=\:\frac{Weight\:of\:dissolved\:solid\:in\:supernatant\times\:100}{Weight\:of\:dry\:solid}$$

#### Scanning electron microscopy (SEM)

The microstructure of the okara samples was investigated by a Scanning Electron Microscope (Tescan, Vega3, Czech Republic). Each sample was coated with gold and then measured at 10.0 kV under 300× and 1000× magnifications.

#### X-ray diffraction (XRD) analysis

The powder samples were filled into the sampler chamber. X-ray diffraction (XRD) patterns were achieved using an X-ray diffractometer (Empyrean, Malvern Panalytical, Netherlands) at 45 kV, 40 mA, and 2θ with a scan angle from 5° to 80°, step width of 0.0131303° 2, and total dwell time of 53.295 s per step.

#### Fourier transform infrared (FTIR) spectroscopy

The powder samples were packed into a chamber and FTIR spectra of the okara powder samples were determined using a Mid Infrared FTIR Spectrometer (Mid FTIR) (Bruker, INVENIO, Germany) over a spectral range of 400–4000 cm^−1^ with 64 scans.

### Determination of chemical properties

The protein total dietary fiber and fat content in okara powder were determined according to AOAC methods^14^ 991.20, 985.29 and 2003.05, respectively.

### Determination of functional properties

#### Sample extraction

The okara powder was extracted by mixing 1 g of okara powder with 10 mL of 80% methanol. The mixture was sonicated for 15 min and centrifuged at 500 rpm for 10 min to separate into clear and gel fractions. The clear solution was used for further antioxidant analysis for both DPPH and ABTS.

#### DPPH assay

Antioxidant activities of the okara cellulose powders were analyzed using the 2, 2-diphenyl-1-picrylhydrazyl (DPPH) assay following the method described by Sensoy et al.^15^. The okara powder was measured for its DPPH radical scavenging ability as the ability of the food product to resist oxidation. One milliliter of the extract solution was mixed with 1 mL of DPPH solution and then vortexed immediately and incubated in the dark at room temperature for 30 min. After incubation, the absorbance of all samples was measured at 515 nm in 1 mL cuvettes using a spectrophotometer (Thermo Scientific, model Genesys 10 S UV-Vis, USA). The results were reported in µmol Trolox equivalent/mL sample.

Working standard solutions were prepared by diluting Trolox solutions at concentrations ranging from 0 to 500 µM. All standard solutions were treated with okara powder before absorbance measurement at a wavelength of 515 nm. The analysis results were calculated using a linear equation based on the calibration curve: y = − 0.0104x + 1.3056 (R² = 0.998), where x is the absorbance and y is the concentration of Trolox.

#### ABTS assay

The antioxidant activity in terms of ABTS radical scavenging capacity was analyzed following the method reported by Klongdee^16^. The ABTS solution (10 mg ABTS in 2.6 mL potassium persulfate (2.45mM) was prepared and stored in the dark at room temperature for 16 h. Before the analysis, an ABTS working solution was prepared by diluting the stock solution in ethanol until the absorbance was in the range of 0.700 ± 0.02 at 734 nm. Samples or standard solution (1 mL) were mixed with 3 mL of ABTS working solution and kept in the dark at room temperature for 5 min. Absorbance was then measured at 734 nm wavelength and radical scavenging activity was expressed as Trolox equivalent antioxidant capacity (µmol Trolox/mg). The analysis results were determined using the following equation based on the calibration curve: y = − 0.0079x + 0.5096 (R2 = 0.998), where x is the absorbance and y is the concentration of Trolox.

### Rheological testing

#### Sample preparation

A modified approach to pre-treating okara powder with NaOH was adopted, based on the procedure outlined by Arai et al.^8^. Two grams of the sample were carefully sieved through a 60-mesh sieve and mixed with 40 mL of 1 M NaOH. This mixture was stirred using a magnetic stirrer and maintained at 60 °C for 30 min before centrifuging at 9800*g* for 20 min under a controlled temperature of 4 °C. The clear portion was then discarded, and distilled water was added to facilitate the removal of the base. This centrifugation process was repeated four times to ensure thorough base removal and neutralization of the sample.

#### Rheological analysis

Dynamic oscillatory shear measurements of the viscoelastic properties of the samples followed those previously reported by Aussanasuwannakul et al.^17^ using a modular compact rheometer (Model MCR 302; Anton Paar, Graz, Austria) with parallel-plate geometry, 25-mm diameter for the upper plate, and gap width of 1 mm. The linear viscoelastic (LVE) range was determined using amplitude sweep tests before starting frequency sweep tests. To understand the structural character of the samples, amplitude sweeps were carried out by implementing a logarithmic increase of the strain (γ) from 0.01 to 100% with six measuring points per decade and a constant angular frequency (ω) of 10.0 rad/s, with temperature set at 25 °C. Storage moduli (G′) and loss moduli (G″) were recorded and analyzed using RheoCompass™ software (Version 1.30, Anton Paar GmbH, Graz, Austria). The LVE range within which G′ and G″ ran parallel to the x-axis was identified. The structural strength of the sample paste was expressed as the G’ value within the LVE range, while the limiting value of the LVE range (γ_L_) was the strain value at which the G′-curve began to deviate from the LVE plateau, with a 5% range of tolerated deviation. The flow point (%) at the crossover point where G′ = G″ was also identified. Frequency sweep tests were conducted at strain = 0.05% between 0.1 and 10.0 rad/s with three measuring points per decade. The gel characteristics of the samples were analyzed according to G′ and G″. Complex viscosity (h*) signified the viscoelastic flow resistance of the sample. A frequency sweep was used to test the stability and performed at 25 °C with ω = 100.0–0.1 rad/s in a predetermined linear viscoelastic range at γ = 0.05% to investigate the time-dependent deformation behaviors of the samples.

### Investigation of specific mechanical energy (SME)

SME indicates the comparison of energy use for production at different conditions. The specific mechanical energy during the extrusion process was calculated from the electrical power used to operate the machine using the following equations^18^.

$$\:\text{SME}\hspace{0.33em}\left(\text{kJ/kg}\right)=\frac{\text{loading}\hspace{0.33em}\text{power}\left(\text{kJ/s}\right)-\text{empty}\hspace{0.33em}\text{power}\left(\text{kJ/s}\right)}{\text{feed}\hspace{0.33em}\text{rate}\left(\text{kg/s}\right)}$$

$$\:\text{SME}=\frac{(\text{Amp}\times\:\text{Volt}{)}_{\text{with}\hspace{0.33em}\text{load}}-(\text{Amp}\times\:\text{Volt}{)}_{\text{with}\hspace{0.33em}\text{no}\hspace{0.33em}\text{load}}}{\text{feed} \:\text{rate}}\hspace{0.33em}$$

where Amp is the electric current recorded from the extruder control panel, and Volt is the voltage recorded from the extruder control panel.

### Experimental design

#### Section 1: studying the effect of process parameters on the physiochemical and functional properties of okara powder

No research has been conducted on this topic; therefore, the effects of factors in the extrusion process were studied as avenues for future experiments. A full factorial design was employed in the experiment to investigate the effect of extrusion parameters on the quality of okara powder. The independent variables and variation levels are listed in Table 1. Each measurement was performed in triplicate, with statistical analysis carried out using MINITAB version 15 statistical software package. One-way ANOVA was used together with Duncan’s post hoc multiple comparison test, with the statistical significance level of the results set at *p* < 0.05.

**Table 1:** Experimental plan to study the production of okara cellulose powder using mechanical energy from the extrusion process.

No.	Screw speed (rpm): SS	Amount of water added (l/h): FM	Number of treatments: T
1	350	0.5	1
2	350	0.5	3
3	350	1.0	1
4	350	1.0	3
5	450	0.5	1
6	450	0.5	3
7	450	1.0	1
8	450	1.0	3

#### Section 2: studying the effect of number of treatment cycles on the physical, chemical and functional properties of okara cellulose powder

The experiment was designed based on the selected conditions from the previous section as determined by the trend of smaller particle sizes and better properties, along with the ability to run the machine. The effect of the number of cycles on the characteristics of the powder was studied, mirroring the research of Ho et al.^5^. The physicochemical and functional properties of the okara cellulose powder and specific mechanical energy were investigated. The quantitative data obtained from each analysis conducted in triplicate were expressed as mean ± SD (standard deviation). One-way ANOVA was used together with Duncan’s post hoc multiple comparison test to analyze the results, with statistical significance set at < 0.05.

## Results and discussion

### Effect of process parameters on the physiochemical and functional properties of okara powder

The extrusion process parameters as screw speed, amount of water added, and number of treatments were investigated. Results in Table 2indicated that the sample treated with retort reduced the mean particle size of the okara powder to 163.2 ± 3.05 microns, while samples treated by heat and mechanical energy from the extrusion process reduced mean particle size ranging between 107.0 and 87.6 microns depending on the extrusion condition. Results revealed that increasing the screw speed of the extruder from 350 to 450 rpm and the amount of water added to the system did not significantly impact the reduction of particle size. However, as the number of treatment cycles increased, particle size decreased, concurring with Ho et al.^5^.

**Table 2:** Physical properties of untreated and treated okara powder.

Sample (SS/FM/T)	Mean particle size (D[3,2]); micron	Swelling ratio	Water solubility index (WSI)
SBM	283.2 ± 10.41 ^d^	4.40 ± 0.77^a^	5.56 ± 0.04^a^
Retort	163.2 ± 3.05^c^	6.88 ± 0.15^d^	8.68 ± 0.57^g^
EX350/0.5/1	103.15 ± 3.02^b^	4.32 ± 0.12^a^	7.87 ± 0.22^c^
EX350/0.5/3	89.78 ± 3.43^a^	5.35 ± 0.54^abc^	7.99 ± 0.19^cd^
EX350/1/1	102.87 ± 2.80^b^	4.37 ± 0.63^a^	7.25 ± 0.21^b^
EX350/1/3	87.6 ± 2.60^a^	5.68 ± 0.27^bc^	8.40 ± 0.10^fg^
EX450/0.5/1	103.51 ± 4.14^b^	4.33 ± 0.61^a^	8.36 ± 0.16^efg^
EX450/0.5/3	91.33 ± 2.38^a^	5.72 ± 0.01^bc^	8.04 ± 0.15^cde^
EX450/1/1	107.0 ± 3.16^b^	4.67 ± 0.06^ab^	8.29 ± 0.03^def^
EX450/1/3	90.83 ± 2.28^a^	5.85 ± 0.03^cd^	8.06 ± 0.10^cdef^

Both forms of mechanical and hydrothermal energy and the extrusion process affected the swelling properties (Table 2). Extrusion at three treatment cycles for every screw speed and amount of water added into the system increased the swelling ratio. The fiber structure changed due to heat and pressure in the hydrothermal process, causing a new structural arrangement called crystalline-to-amorphous transformation^19^and the structure changed from tightly packed to more open caused by the shear force obtained from mechanical energy during extrusion cooking, similar to the shear force from the wet grinding process, as also reported by Ullah et al.^1^. The structure was able to hold more water. Okara powder also contains some starch. When starch is processed using heat and pressure, its structure changes and the water-swelling ability increases upon gelatinization^20^. Thus, when okara powder containing starch was subjected to the extrusion process, a similar reaction occurred, resulting in a higher swelling ratio.

The water solubility index (WSI) values in Table 2indicated that both the retort and extrusion processes tended to increase the WSI of okara samples, related to the reduction of particle size. When particle size decreases, the WSI value increases due to an increase in porosity and capillary attraction, which results in a rearrangement of the insoluble form into a water-soluble form^1^. The WSI is also related to solubilized molecules which indicate the amount of starch conversion that occurs when starch molecules are destroyed and can bind more water^21^. Water solubility is impacted by conditions during extrusion that involve high shear rates and high heat. Low moisture levels of raw materials cause higher temperatures which result in hydrogen bonds being broken. The starch particles are destroyed and further broken down, thereby increasing the water solubility of the product and concurring with results reported by Brennan et al.^22^, Hagenimana et al.^23^ and Singh et al.^24^. Thus, samples treated by the extrusion process had higher WSI values than SBM.

**Fig. 2:**
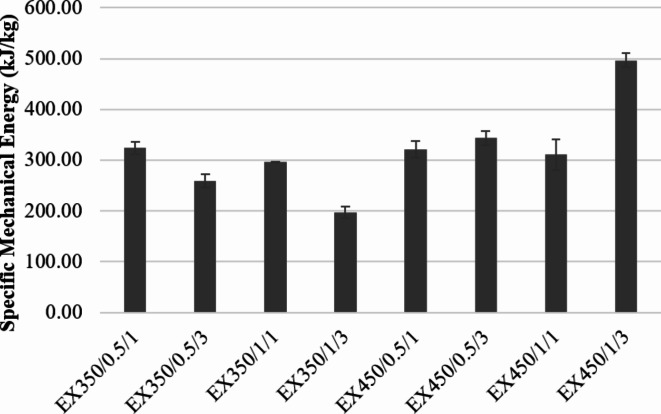
SME values of okra powder under different extrusion conditions.

Specific mechanical energy (SME) measures the energy used in the extrusion process. The SME is only part of the data and not the total amount of energy used in the process; therefore it does not indicate the efficiency of the machine^25^. In the extrusion process, the SME value is a function of many factors including feed moisture, screw speed, and the viscosity of the raw materials. As shown by the experimental results, the SME value ranged between 150 and 500 kJ/kg (Fig. 2). At a screw speed of 350 rpm, the SME value tended to decrease as the number of treatment cycles increased and as the amount of water fed into the system increased. The SME value also decreased as the feed moisture increased because the combined effect of the amount of water and fat in okara powder reduced the viscosity of the raw materials that were transformed into dough inside the extruder. Therefore, this reduced the effects of internal shear forces and the resistance force from rotating the screw, thereby decreasing the torque and also the SME value. This result concurred with Bhattacharya and Hanna^26^and Altan and Maskan^27^. The SME value decreased when the number of treatment cycles increased at a screw speed of 350 rpm because the structure of the raw material such as protein and starch was destroyed. The fat extracted from the raw materials caused the viscosity to decrease, resulting in a decrease in the mechanical energy required to drive the screw and causing the SME value to decrease. However, when increasing the screw speed from 350 to 450 rpm, the SME increased because increasing the screw speed increased the shear force of the material inside the screw, causing higher friction between the barrel wall and the material^28–30^. In particular, increasing the number of cycles to 3 (at a screw speed of 450 rpm and 1 L per hour of water added in the system), the sample began to burn as the structure of both protein and starch was exposed to high shear heating.

Based on these results, extrusion at a screw speed of 350 rpm and 1 L of water added per hour was selected to study how increasing the number of treatment cycles impacted the physical, chemical, and functional properties of okara cellulose powder. This condition was selected because the sample treatment cycles could be increased without burning while the SME value remained in the low range, indicating that the okara powder could reenter the extruder with no interruptions (normally, if the SME values increase, this can cause the sample to burn, with blockage at the die). The physical properties of this condition, in terms of particle size reduction, increased the swelling ratio with higher WSI.

### Effect of number of treatment cycles on the physical, chemical and functional properties of okara cellulose powder

Results showed that the extrusion cycles could be increased to a maximum of 6. When entering the 7th cycle, the sample was burned and blocked in the extruder die. This occurred because samples that contained proteins and starch that were exposed to heat and shear force many times suffered structural change and burned. Therefore, only 6 cycles of treatment were performed.

**Fig. 3:**
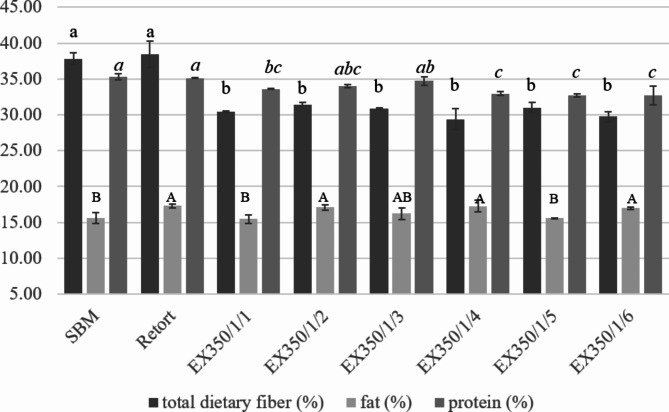
Total dietary fiber, fat and protein content of untreated and treated okara powder at different processes and conditions. Different lowercase letters represent the comparison of total dietary fiber (%), different uppercase letters show the comparison of % fat and different italic lowercase letters represent the comparison of % protein.

Results of the compositional analysis of okara powder (Fig. 3) indicated that the protein and fat content did not change much when comparing the SBM, retort, and extruded samples. However, the amount of dietary fiber in samples that had undergone the extrusion process was significantly reduced compared with SBM and retort. This occurred because the mechanical energy from the extrusion process caused changes in the structure of the food fibers, resulting in increased solubility^31^. Chen et al.^4^ found that okara powder which had undergone ball milling at high speed showed reduced insoluble dietary fiber because the extrusion treatment transformed insoluble dietary fiber to soluble fiber.

**Fig. 4:**
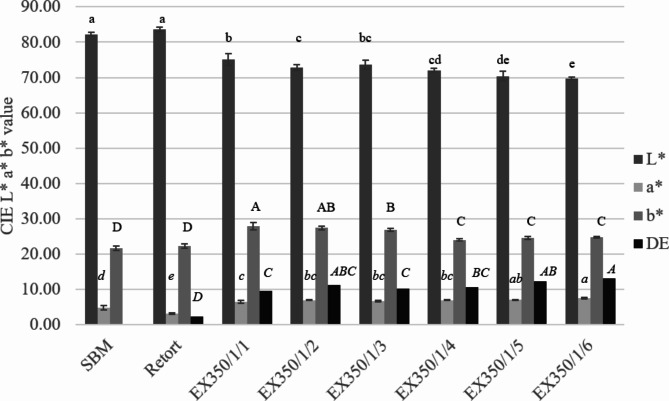
Color of untreated and treated okara powder at different processes and conditions. Different lowercase letters represent the comparison of L*, different italic lowercase letters show the comparison of a*, different italic uppercase letters represent the comparison of b* and different italic uppercase letters show the comparison of ΔE.

**Fig. 5:**
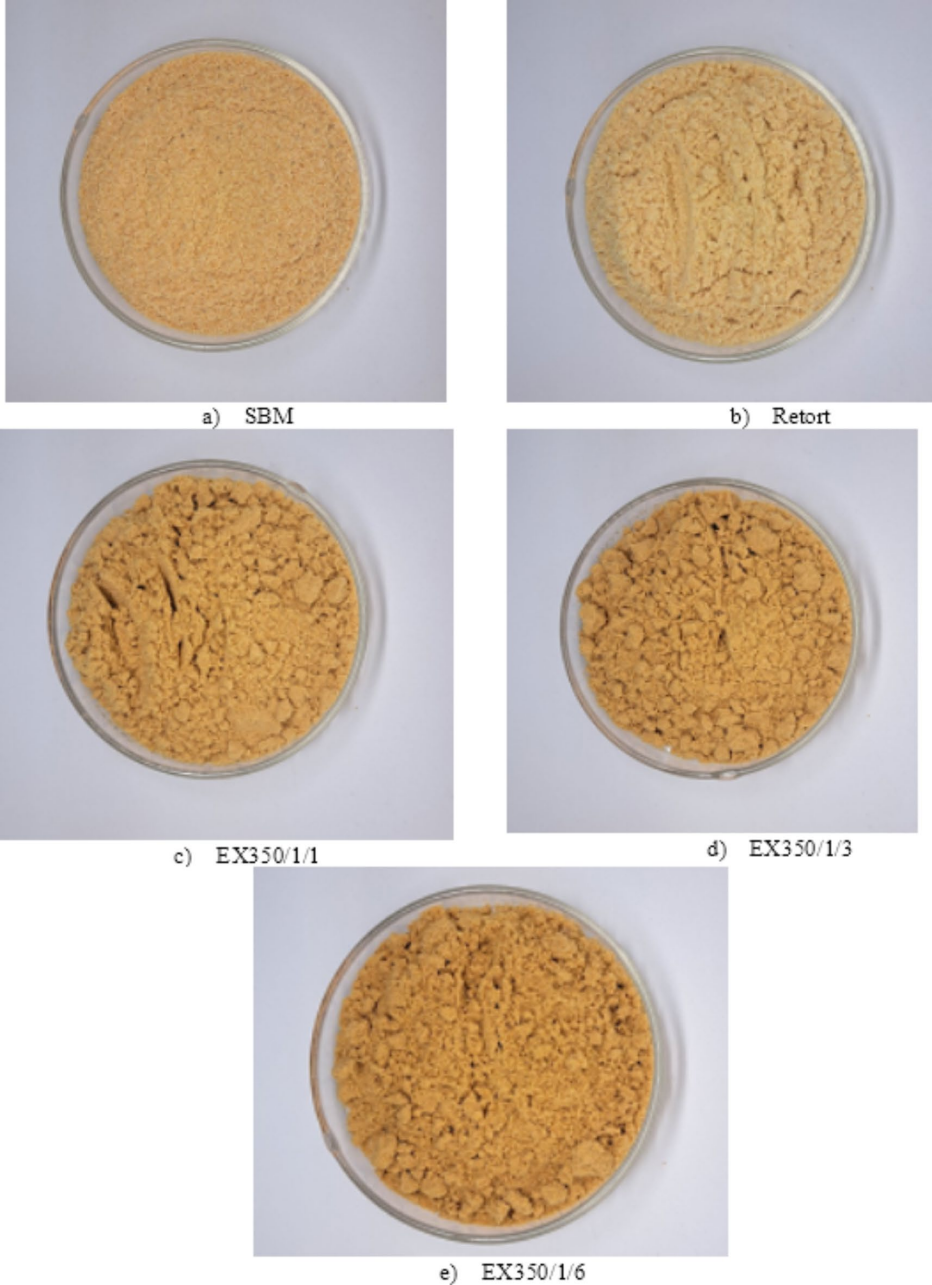
Untreated okara powder (SBM) and treated okara powder at different processes and conditions.

The effect of the number of passes through the extrusion process on the color value is shown in Fig. 4. The color of the product was measured using the L*, a*, b* color system where ΔE, L*, a*, and b* values represent color difference, darkness-lightness, green-red and blue-yellow values, respectively. The L* and b* values decreased with an increase in the number of treatment cycles. The a* value increased because samples subjected several times to higher heat with low moisture became darker (as shown in Fig. 5) due to the Maillard reaction during extrusion. A similar occurrence in the milling process was also reported by Chen et al.^4^. The ΔE value tended to increase when increasing the number of treatment cycles as expected, while the ΔE value of the retorted samples was less than for the extrusion samples because a higher moisture content in the retort process helped to preserve the color of the sample.

**Fig. 6:**
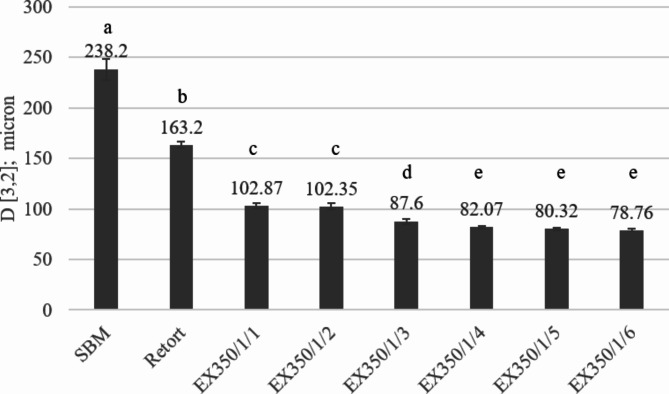
Particle size of okara powder samples treated with different processes and conditions. Different lowercase letters show the significant differences among the treatments in particle size.

The effect of the number of treatment cycles on particle sizes of okara powder is presented in Fig. 6. When comparing the SBM with the samples that were mechanically processed, both the retort method and the extrusion process (at screw speed of 350 rpm with water fed into the system of 1 L/h, feeding into the extruder for 1–6 cycles) providing mechanical energy reduced the particle size of the okara powder. Increasing the number of treatment cycles during extrusion gave smaller particle sizes than the retort method. Particle size reduced to 78.76 ± 1.35 microns after 6 treatment cycles, concurring with Ho et al.^5^.

**Fig. 7:**
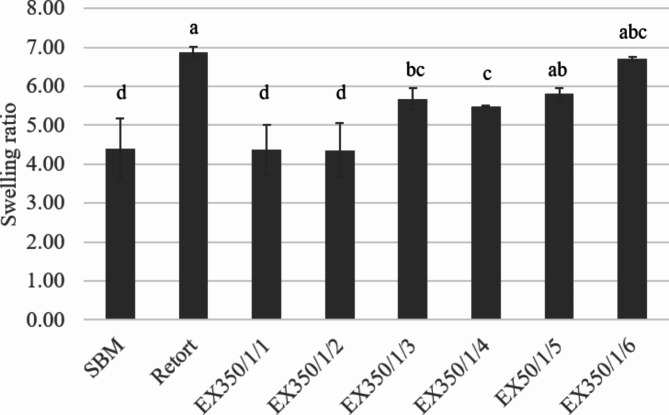
Swelling ratio of okara samples treated with different processes and conditions. Different lowercase letters present the significant differences among the treatments in swelling ratio.

The results of the swelling ratio are shown in Fig. 7. Increasing the number of cycles of treating soybean meal with the extrusion process increased the swelling ratio because the structure of the fibers changed. The compacted tissue becomes an open structure due to the shear force received from mechanical energy, resulting in the structure being able to hold more water. Similarly, the shear force from the wet grinding process increased the swelling properties^1^. More heat and pressure caused the structure of the okara powder to change and increased the water-swelling ability^20^.

**Fig. 8:**
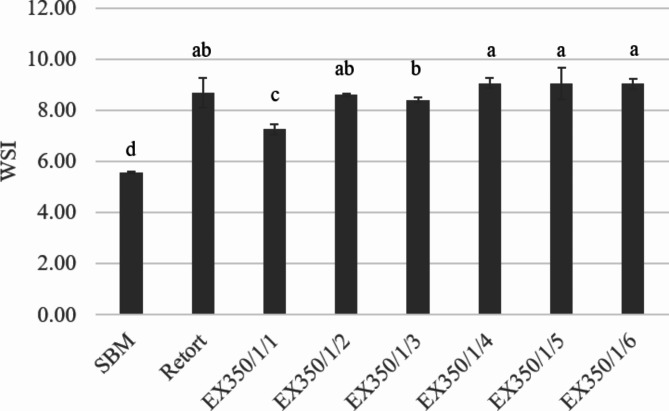
WSI of okara powder samples treated with different processes and conditions. Different lowercase letters represent the significant differences among the treatments in WSI.

The water solubility index (WSI) values (Fig. 8) showed that increasing the number of sample treatment cycles with the extrusion process tended to increase the water solubility of the soybean meal samples. This occurred because of the reduction of particle size and the structural changes of fiber and starch, as already described in “Effect of process parameters on the physiochemical and functional properties of okara powder”.

**Fig. 9:**
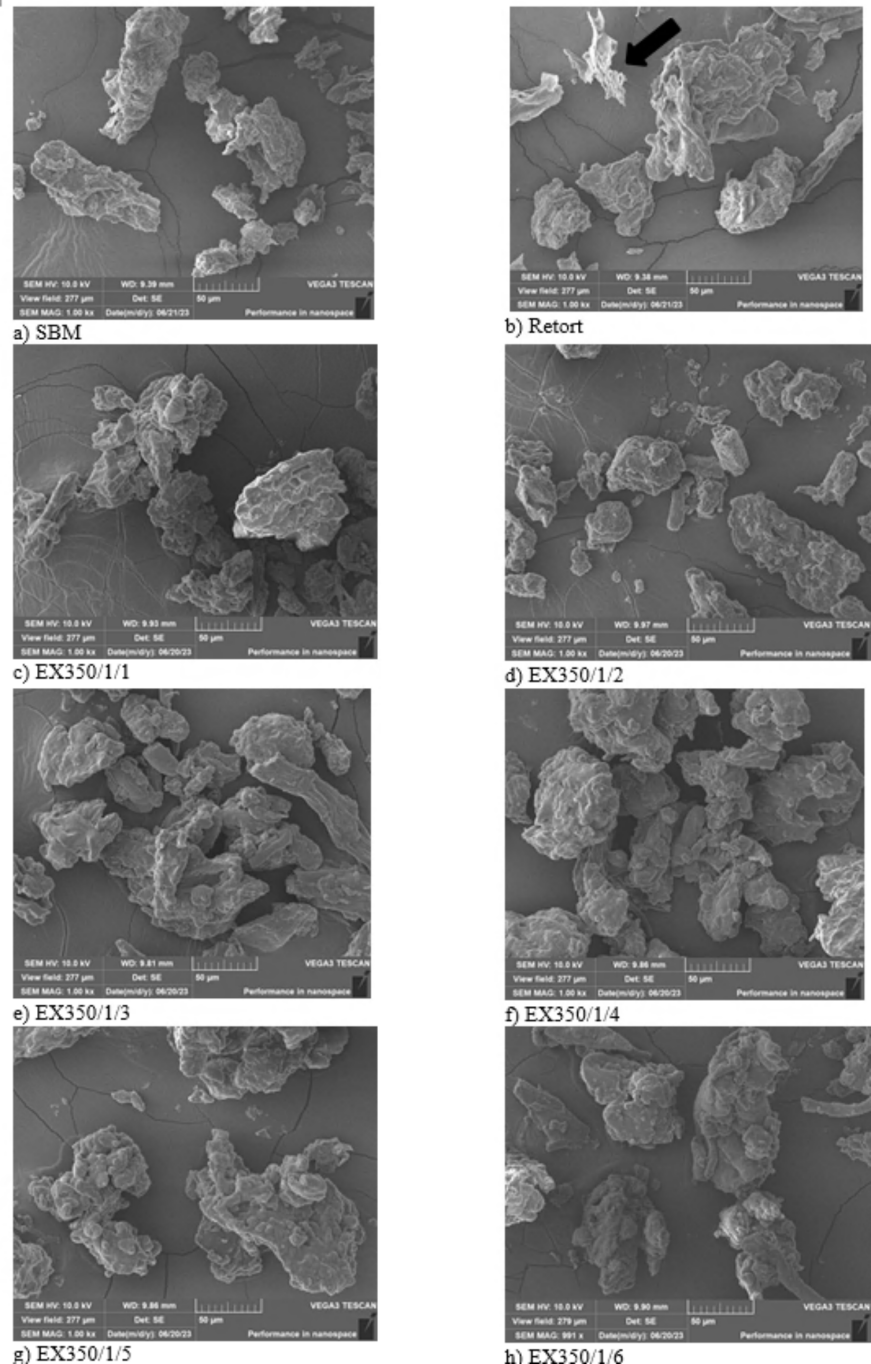
Scanning electron microscopic images of okara powder samples.

The microstructures of okara cellulose powder are shown in Fig. 9. The surface of the untreated sample (SBM) and retorted sample were uneven and compact with some lesions at the edges, especially for the retort sample, as shown by the arrow, because of the heat and pressure generated from the retort process. For the extrusion process at screw speed 350 rpm, the amount of water added into the system at 1 L per hour and at 1–6 cycles of treatment showed that the microstructure had similar characteristics, with a smoother surface than SBM and retort. The lesions and sharp corners disappeared, consistent with Ullah et al.^1^ who processed okara powder using a wet grinding process to reduce particle size. The surface of the samples became smoother because of the shear force from crushing during grinding. Agglomeration also occurred in samples that had undergone more extrusion treatment cycles because fat increased as the number of treatment cycles increased. The fat was extracted from the structure and stuck to the surface. Uitterhaegen and Evon^32^ utilized the extrusion process to extract oil from various grains. They determined that oil could be extracted on an industrial scale using extrusion and promoted as a green technology.

**Fig. 10:**
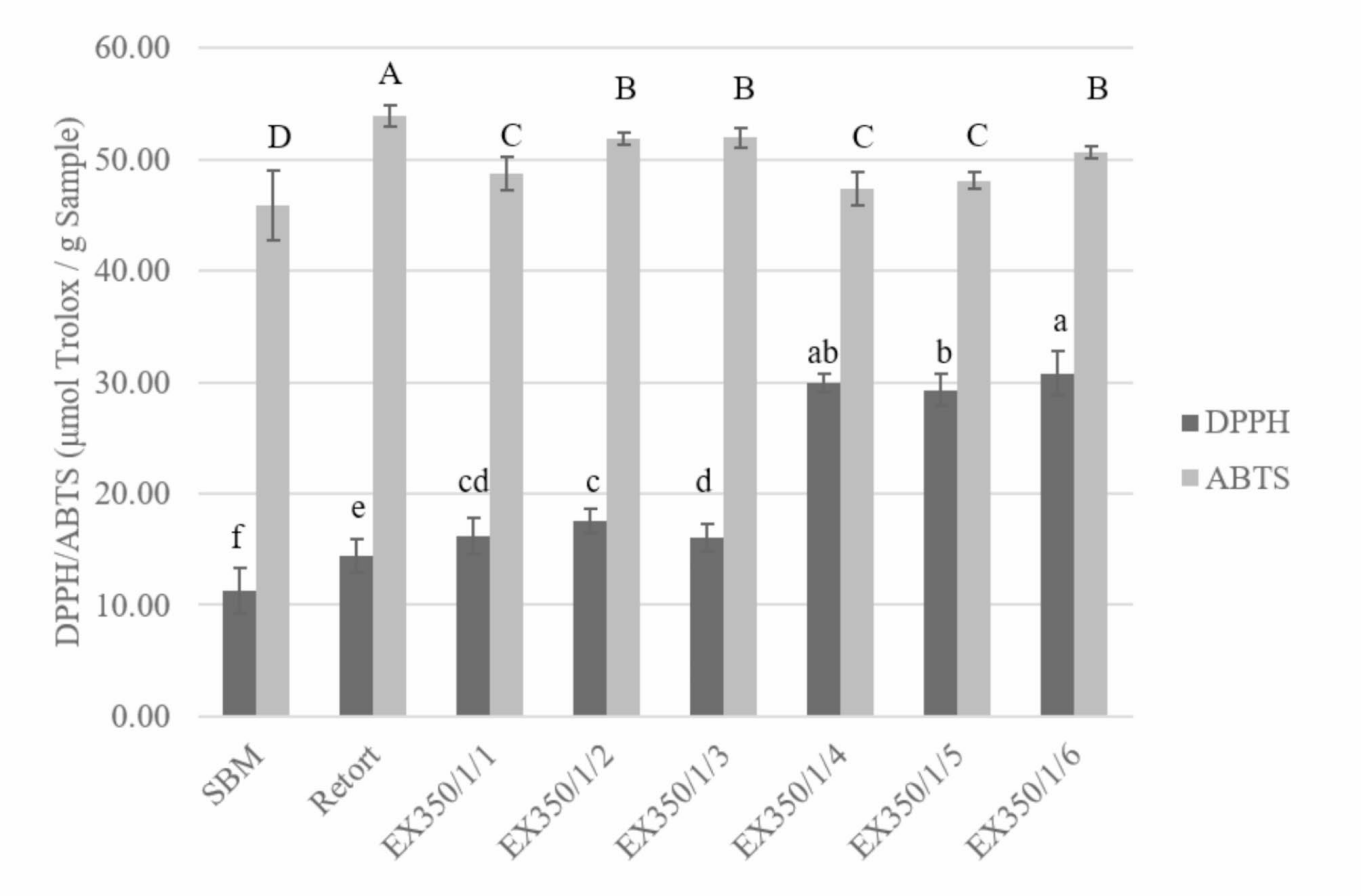
DPPH and ABTS antioxidant capacity of okara powders treated with different processes and conditions.

The antioxidant abilities in terms of the DPPH radical scavenging activity and the ABTS radical cation decolorization assay were also determined. Results showed higher antioxidant capacity when the okara powders were subjected to extrusion and hydrothermal processes, as shown in Fig. 10, caused by the reaction of Maillard browning pigments that promoted antioxidant capacity^33^. An increase in antioxidant capacity was also observed when the samples were processed through the extrusion process, as reported by Hu et al.^10^ and Acosta et al.^34^. Increasing the number of cycles in the extrusion treatment also increased the antioxidant capacity in the DPPH method while the ABTS method showed an increase in 1–3 rounds and then decreased in the next round because the heat from the process destroyed some of the antioxidants.

**Fig. 11:**
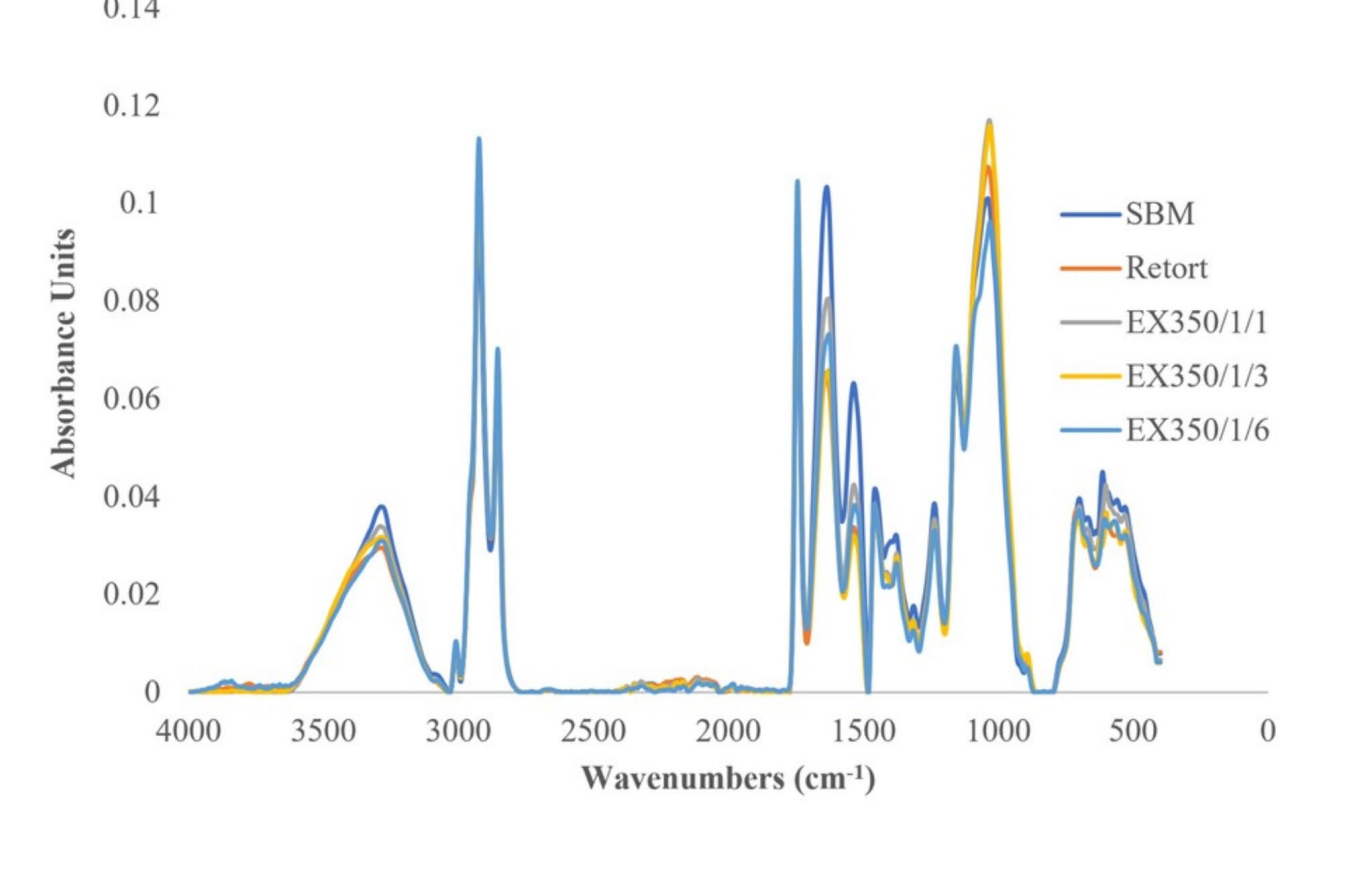
Fourier transform infrared spectroscopy (FTIR) spectra of okara cellulose powder in different treatments.

The FTIR spectra of okara cellulose powder, both untreated and treated, are presented in Fig. 11. Overall, the graph characteristics were not different among SBM, retort and extrusion. The hydrothermal (retort) and mechanical treatment methods from the extrusion process did not affect the bioactive compounds of okara powder. Changes in the peak intensities were detected, indicating that the characteristic functional groups of okara powder were not destroyed by mechanical treatments (heat, pressure, and shear) under both hydrothermal and extrusion processes. This result concurred with other mechanical treatments and intense disruptive forces reported by Chen et al.^4^ and Wu et al.^3^. All the spectra showed the classical structure of cellulose or hemicellulose, with bands from 3000 to 3700 cm^−1^ and the peak at 2923 cm^−1^representing O–H stretching vibration groups and the C–H bond in the methyl group^3,4^. The peak at about 1040 was attributed to stretching of the carbonyl group (C–O) and CC-band, indicating that the insoluble dietary fiber (IDF) decomposed into oligosaccharides^3,4^. The peaks at 891–897 cm^−1^were attributed to bending vibrations of the C–1 group and related to the amorphous region of cellulose^3,4^, while the peaks observed at 1532–1536 cm^−1^ suggested that the okara cellulose powder contained protein^3^, concurring with the protein reported in Fig. 3.

**Fig. 12:**
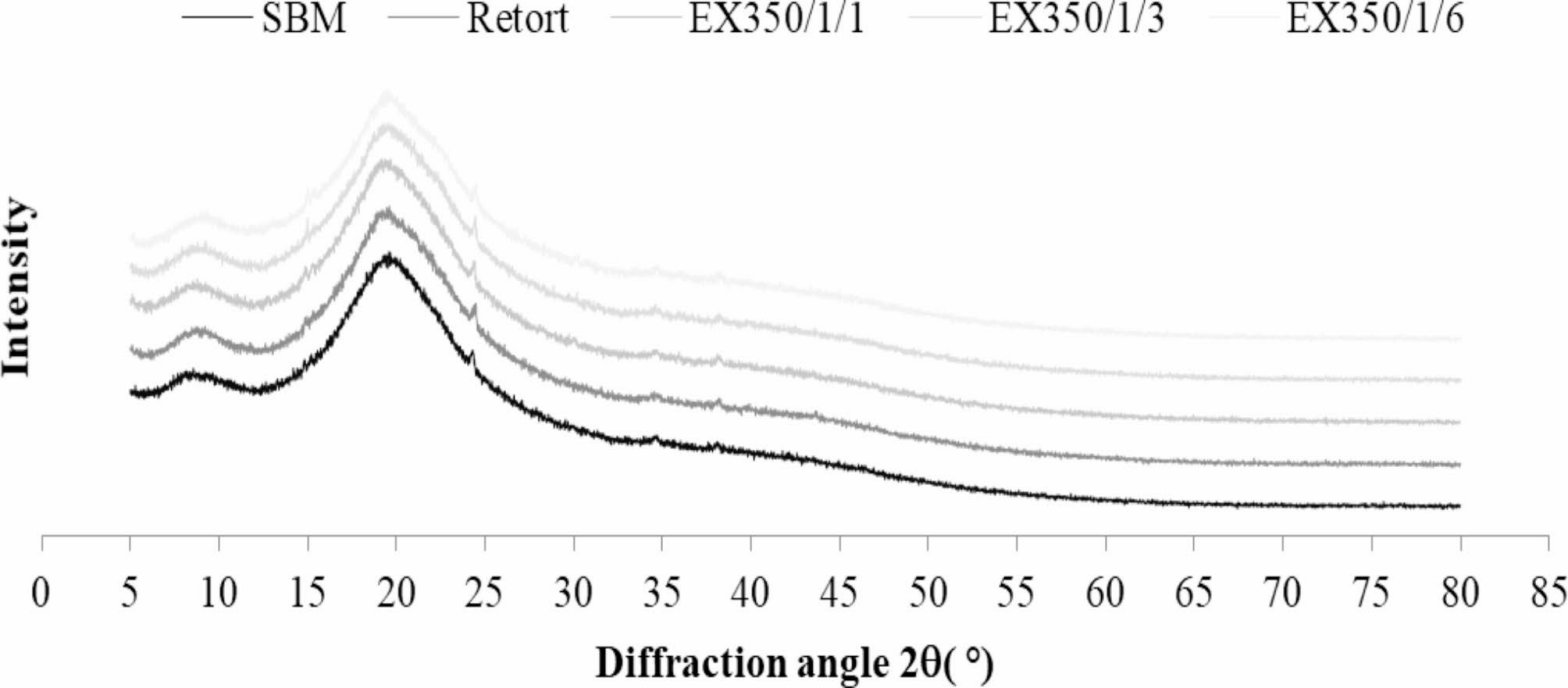
XRD Patterns of untreated and treated okara powder.

Figure 12shows the XRD patterns of untreated and treated okara powder. Results indicated that the peaks were maintained after all the mechanical treatments, with no significant impact on the crystalline structure. The XRD patterns showed that the typical peaks of cellulose between 2θ = 12°–24° broadened to a single peak. The amorphous region inhibited the crystalline region peaks because various amorphous components in the okara powder such as proteins, ash, and small molecules increased the amorphous region in the XRD patterns. This result concurred with Chen et al.^4^ who used the ball milling method to modify the soybean residue, while Camargo et al.^35^ worked with wet and dry ball milling to reduce the size of alumina powder.

**Fig. 13:**
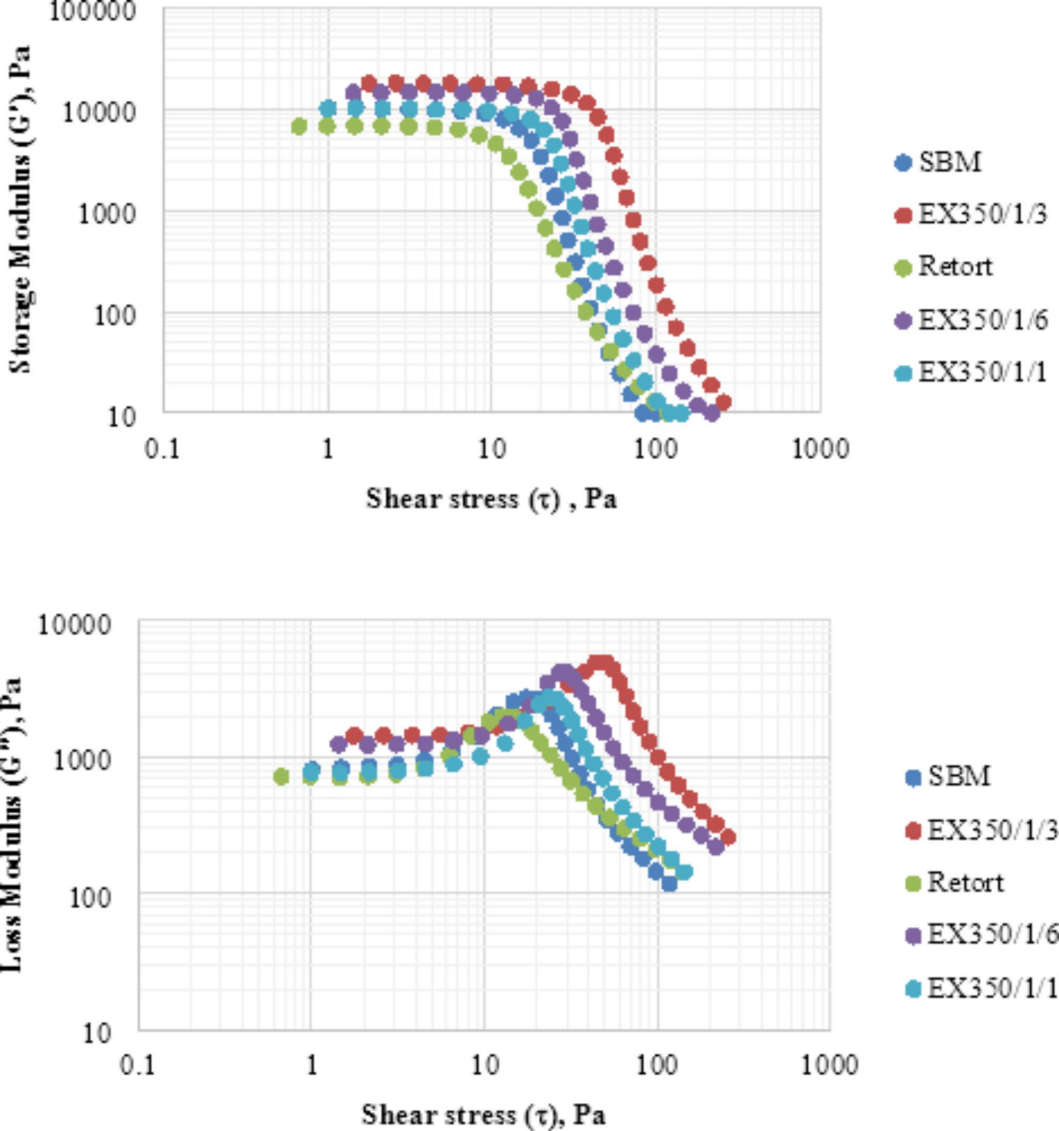
Amplitude sweep test of untreated and treated samples.

**Fig. 14:**
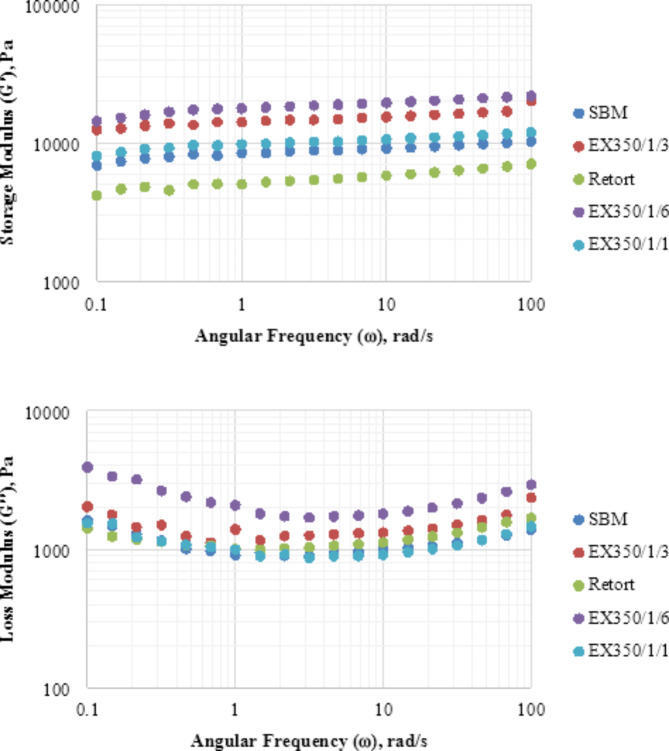
Frequency sweep test of untreated and treated samples.

The rheological properties of SBM retort and extruded at 350/1/1, 350/1/3, and 350/106 using dynamic oscillatory shear and analyzed in terms of amplitude sweep and frequency sweep are shown in Figs. 13 and 14. Results of the amplitude sweep test indicated that at shear stresses lower than 10 Pa, the storage modulus (G′) values remained the same but the loss modulus (G″) values increased with increasing shear stress. Samples treated with extrusion had higher G′ and G″ values than those treated by SBM and retort because they had a stronger structure. Extrusion treatment caused changes in particle size, morphology, and flexibility with increasing sample viscosity. This result concurred with Huang et al.^36^ who studied the effect of heat treatment using a high-pressure homogenizer on the rheological properties of soy protein isolate gels. When the shear stress increased to more than 10 Pa, the storage modulus G′ and loss modulus G″ gradually decreased with increasing shear stress, indicating that the bonds in the gel network were broken and the samples deteriorated.

In the frequency sweep test, G′ and G″ values increased when the number of extrusion treatments increased. The gel became more elastic and the network structure strengthened as the gel formed larger aggregates. Extrusion treatment enhanced the elasticity and viscosity of the gel due to increased cellulose content and strengthened the hydrogen bonds^36^. For the retort sample, the G′ value was lower than the other samples, indicating that gel samples from retorted soybean meal powder had the most easily broken structure. Graphical results showed G′ values higher than G″, indicating that all okara powders have dominant elastic properties characteristic of a solid structure^17^.

**Fig. 15:**
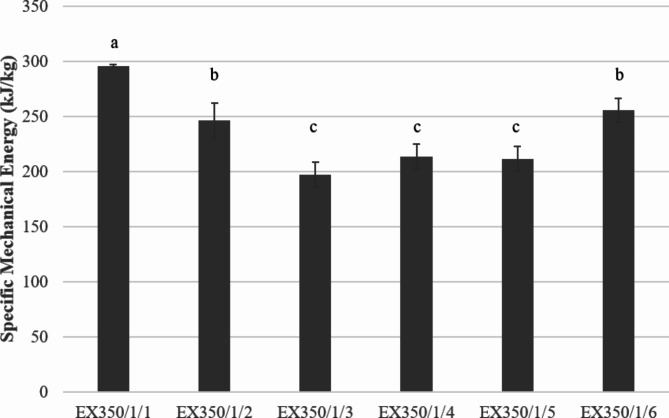
Effect of the number of extrusion treatments on SME.

The SME value decreased when increasing the number of treatment cycles in the first 1–5 cycles, as shown in Fig. 15. The structures of the raw materials such as proteins and starches were destroyed while the fat extracted from the raw materials caused the viscosity to decrease, resulting in a decrease in the mechanical energy required to drive the screw. However, when increasing the number of cycles to 6, the SME value decreased, indicating that the sample was affected by heat, as evidenced by the darker color.

## Conclusions

Results proved that the extrusion process significantly reduced the particle size of okara powder and improved both physical and functional properties. Treating okara powder by extrusion at a screw speed of 350 rpm, with water added at 1 L per hour and 6 cycles of treatment, reduced particle size to 78.76 ± 1.35 microns. Samples treated by both hydrothermal and mechanical extrusion methods gave increased swelling ratios and WSI but only the extrusion treatment improved the rheological properties. Higher numbers of treatments increased the swelling ratio and WSI values, while antioxidant capacity increased when the okara powder was subjected to extrusion and hydrothermal processes. The color of the okara powder became darker when processed at a higher number of extrusion treatment cycles. The extrusion process was an effective mechanical method that improved the physical and functional properties of okara cellulose powder by converting the by-product into a value-added ingredient for use in future food applications. However, this process is limited and cannot be used with raw materials that have high fat and protein contents. High fat content can cause slipping during the process, while high protein content can cause the product to burn. Future studies may need to incorporate chemical pre-treatment during the extrusion process to reduce the size of the okara powder.

## Data Availability

The data that support the findings of this study are available from the corresponding author upon reasonable request.
